# Profiles of white matter tract pathology in frontotemporal dementia

**DOI:** 10.1002/hbm.22468

**Published:** 2014-02-07

**Authors:** Colin J. Mahoney, Gerard R. Ridgway, Ian B. Malone, Laura E. Downey, Jonathan Beck, Kirsi M. Kinnunen, Nicole Schmitz, Hannah L. Golden, Jonathan D. Rohrer, Jonathan M. Schott, Martin N. Rossor, Sebastien Ourselin, Simon Mead, Nick C. Fox, Jason D. Warren

**Affiliations:** ^1^ Dementia Research Centre UCL Institute of Neurology University College London London United Kingdom; ^2^ Wellcome Trust Centre for Neuroimaging UCL Institute of Neurology University College London London United Kingdom; ^3^ MRC Prion Unit UCL Institute of Neurology University College London London United Kingdom; ^4^ Centre for Medical Imaging Computing UCL University College London London United Kingdom

**Keywords:** frontotemporal dementia, DTI, tract, tractography, white matter

## Abstract

Despite considerable interest in improving clinical and neurobiological characterisation of frontotemporal dementia and in defining the role of brain network disintegration in its pathogenesis, information about white matter pathway alterations in frontotemporal dementia remains limited. Here we investigated white matter tract damage using an unbiased, template‐based diffusion tensor imaging (DTI) protocol in a cohort of 27 patients with the behavioral variant of frontotemporal dementia (bvFTD) representing both major genetic and sporadic forms, in relation both to healthy individuals and to patients with Alzheimer's disease. Widespread white matter tract pathology was identified in the bvFTD group compared with both healthy controls and Alzheimer's disease group, with prominent involvement of uncinate fasciculus, cingulum bundle and corpus callosum. Relatively discrete and distinctive white matter profiles were associated with genetic subgroups of bvFTD associated with *MAPT* and *C9ORF72* mutations. Comparing diffusivity metrics, optimal overall separation of the bvFTD group from the healthy control group was signalled using radial diffusivity, whereas optimal overall separation of the bvFTD group from the Alzheimer's disease group was signalled using fractional anisotropy. Comparing white matter changes with regional grey matter atrophy (delineated using voxel based morphometry) in the bvFTD cohort revealed co‐localisation between modalities particularly in the anterior temporal lobe, however white matter changes extended widely beyond the zones of grey matter atrophy. Our findings demonstrate a distributed signature of white matter alterations that is likely to be core to the pathophysiology of bvFTD and further suggest that this signature is modulated by underlying molecular pathologies. *Hum Brain Mapp 35:4163–4179, 2014*. © **2014 The Authors. Human Brain Mapping Published by Wiley Periodicals, Inc.**

## INTRODUCTION

The behavioral variant of frontotemporal dementia (bvFTD) is a major neurodegenerative syndrome of younger life [Ratnavalli et al., 2002]. It is the most common of the canonical syndromes of frontotemporal lobar degeneration. The syndrome is led by progressive decline in personality and inter‐personal skills with emergence of a range of behavioral abnormalities including obsessionality, loss of empathy and apathy [Rascovsky et al., 2011]. Most cases are associated with pathology in the frontotemporal lobar degeneration (FTLD) spectrum, however pathological substrates are heterogeneous [Rohrer et al., 2011; Seelaar et al., 2011]. The neuroimaging features of bvFTD are also variable: typically, there is cortical atrophy involving the frontal and temporal lobes, often asymmetric between the cerebral hemispheres [Rohrer et al., 2011; Whitwell and Josephs, 2011]. Despite much recent interest, prediction of tissue pathology from clinical and neuroanatomical features in bvFTD remains problematic [Seelaar et al., 2011], yet this is likely to be crucial both for understanding the neurobiology of bvFTD and for the evaluation and monitoring of future disease‐modifying therapies. The concept of network‐led neurodegeneration offers a potentially unifying neurobiological basis for the diverse spectrum of clinical and neuroimaging phenotypes produced by pathogenic proteins [Raj et al., 2012; Zhou et al., 2012]. Network disintegration in bvFTD has been demonstrated using functional MRI and distributed grey matter changes compatible with network breakdown have been established [Rohrer et al., 2010; Seeley et al., 2009; Whitwell and Josephs, 2011; Whitwell et al., 2005, 2012]. In particular, an anteriorly directed “salience” processing network including anterior cingulate, insula and prefrontal cortices has been implicated in the pathogenesis of bvFTD [Seeley et al., 2009]. However, there remains a paucity of information on the changes occurring within white matter tracts, which provide structural connectivity underpinning large‐scale brain networks by binding together cortical and subcortical regions. Furthermore, besides common syndromic profiles of network damage there may be distinct neuroanatomical subgroups within the bvFTD spectrum with relative specificity for particular molecular pathologies [Warren et al., 2012]. Identifying profiles of white matter pathway damage along molecular lines in bvFTD would both validate the concept of network‐led degeneration and potentially allow additional (or more robust) stratification of neuroanatomical subgroups within the bvFTD spectrum that might map more closely onto pathological and genetic substrates.

The comparatively recent advent of diffusion tensor imaging (DTI) allows such white matter tract profiles to be delineated. A particular potential strength of DTI is the generation of multiple quantitative metrics of white matter alteration (diffusivity) during a single acquisition. The neurobiological significance of these metrics remains relatively little explored: whilst fractional anisotropy (FA) has been the most widely cited metric, more recent studies have suggested that use of the individual parameters of diffusion may be more appropriate in neurodegeneration [Acosta‐Cabronero et al., 2010; Mahoney et al., 2013]. Further it has been suggested that particular DTI metrics such as axial diffusivity (AX) radial diffusivity (RD) and trace diffusivity (TR) may have specificity for particular pathologies [Nair et al., 2005; Song et al., 2002] although this remains controversial [Jones et al., 2013; Wheeler‐Kingshott and Cercignani, 2009]. Previous studies (see Table 1) of white matter tract damage in bvFTD have been relatively small and there is limited information about tract alterations associated with particular bvFTD pathologies and how these compare with other common neurodegenerative disorders such as Alzheimer's disease (AD). Often only FA has been assessed, potentially anatomically biased ‘seed‐based’ approaches have been used or technical factors such as normalisation‐associated distortion have not been fully addressed [Agosta et al., 2011; Whitwell et al., 2010; Zhang et al., 2009, 2013]. More fundamentally, it remains unclear how white matter alterations relate to the more familiar profiles of grey matter atrophy in bvFTD.

**Table 1:** Comparison of fractional anisotropy changes in studies of frontotemporal dementia using diffusion tensor imaging

Study	Participants	MRI acquisition	Software	Tract selection	Statistical method	Tracts
Matsuo et al., 2008	12 bvFTD, 17 Controls	1.5 Tesla, 15 directions	PRIDE	ROI	Mann‐Whitney *U*	AF, UF, ILF, CC
Zhang et al., 2009	18 bvFTD; 18 AD; 19 Controls	4 Tesla; 6 directions	Volume‐one/dTV	Whole brain, ROI	MANOVA/multiple regression	Anterior CC, UF, anterior/descending/left posterior CB
Whitwell et al., 2010	16 bvFTD; 19 Controls	3 Tesla; 21 directions	DTI Studio	ROI	ANCOVA	Anterior CB, UF, SLF, right anterior ILF, right CST
Agosta et al., 2011	13 bvFTD; 25 Controls	3 Tesla; 32 directions	FDT/TBSS	Whole brain, ROI	Permutation test (*n* = 5,000)	CB, Fornix, CC, SLF, UF, ILF, Parahippocamal tract
Lillo et al., 2012	15 bvFTD; 18 Controls	3 Tesla; 32 directions	FDT/TBSS	Whole brain	Permutation test (*n* = 5,000)	Anterior CC, anterior ILF, CST
Zhang et al., 2013	13 bvFTD; 19 Controls	4 Tesla; 6 directions	SPM8	Whole brain, ROI	Two‐sample *t*‐test	UF, anterior CC
Present study	27 bvFTD; 25 AD; 20 Controls	3 Tesla; 64 directions	Camino/TBSS	Whole brain, ROI	Permutation test (*n* = 10,000)	UF, CB, CC, SLF, ILF, ATR, CST

Tracts listed are bilateral unless otherwise stated. A statistical threshold of *P* <0.05 was used for citing tracts with significant change from individual papers. AD, Alzheimer's disease; ANCOVA, analysis of covariance; AF, Arcuate Fasciculus; ATR, anterior thalamic radiation; CB, cingulum bundle; CC, corpus callosum; Controls, older healthy control participants; CST, corticospinal tract; DTI, diffusion tensor imaging; FDT, FMRIB diffusion toolbox; bvFTD, frontotemporal dementia; ILF, inferior longitudinal fasciculus; MANOVA, Multivariate analysis of variance; PRIDE, Philips Research Integrated Device Environment; ROI, region of interest; SLF, superior longitudinal fasciculus; SPM8, Statistical Parametric Mapping version 8; TBSS, tract based spatial statistics; UF, uncinate fasciculus.

In this study, we set out to define in detail profiles of white matter tract damage using DTI in a comprehensively characterized cohort of patients with bvFTD representing major genetic as well as sporadic forms, in relation both to healthy control individuals and to patients with AD. We hypothesized that separable profiles of white matter pathology would be identified within this broad bvFTD spectrum and in comparison to AD. Subsidiary aims of our study were to compare the profiles of different diffusivity metrics in bvFTD, using anatomically unbiased tract‐based and customised dementia template‐based techniques; and to compare white matter alterations in relation to the distribution of grey matter atrophy.

## METHODS

### Participants

Twenty‐seven consecutive patients who fulfilled a diagnosis of probable or definite bvFTD based on current consensus criteria [Rascovsky et al., 2011] were recruited from the Specialist Cognitive Disorders Clinic at the National Hospital for Neurology and Neurosurgery. The bvFTD cohort included 14 patients with either neuropathologic confirmation or pathogenic mutations in the *MAPT* or *C9ORF72* genes, three subjects with *C9ORF72* have previously had their neuroimaging findings reported elsewhere [Mahoney et al., 2012a]. Twenty healthy older volunteers and 25 patients with a diagnosis of probable AD [Dubois et al., 2007] also participated. Participants underwent a structured clinical and neuropsychological assessment (see Supporting Information for detailed information on tests used) and structural magnetic resonance imaging (MRI) to exclude significant vascular disease or other focal cerebral lesions. Demographic and neuropsychological data were analyzed in STATA12® (Statacorp) using Student's *t* test and Wilcoxon rank‐sum tests of statistical significance.

Ethical approval for the study was obtained from the local institutional ethics committee and all participants gave written informed consent to participate in accordance with the Declaration of Helsinki.

### Genetic Analysis of Subjects

All 27 patients were asked to contribute a DNA sample for genetic analysis; one patient declined and one patient later withdrew consent for testing; both of these patients had no family history and were therefore considered to have sporadic disease. The remaining 25 patients underwent genetic sequencing of *MAPT, PGRN, or C9ORF72* using either direct Sanger sequencing or with next generation sequencing technology using Life Technology's Ion Torrent Personal Genome Machine sequencing; which additionally screened for mutations within fused‐in‐sarcoma, valosin‐containing protein, charged multivesicular body protein 2B, prion protein, TAR DNA‐binding protein 43, presenilin 1 and 2, amyloid precursor protein and colony stimulating factor 1 receptor genes.

### Acquisition of Brain Images

Brain MRI data were acquired for all participants on a Siemens Trio 3T MRI scanner using a 32‐channel phased array head‐coil (Siemens, Erlangen, Germany). Two 64‐direction DTI sequences were acquired with a single shot, spin‐echo echo planar imaging sequence (55 contiguous axial 2.5 mm slices with 240 mm field of view and 96 × 96 matrix, yielding 2.5 mm isotropic voxels; repetition time: 6,800 ms; echo time: 91 ms; b value: 1,000 s/mm^2^), augmented with parallel imaging acceleration to reduce susceptibility artifacts. Nine images without diffusion weighting were also acquired (*b* = 0 s/mm^2^). Other sequences acquired were a sagittal three‐dimensional magnetization‐prepared rapid gradient echo T1‐weighted volumetric MRI (echo time/repetition time/inversion time = 2.9/2,200/900 ms, dimensions of 256 × 256 × 208, voxel size of 1.1 × 1.1 × 1.1 mm) and a coronal fluid‐attenuated inversion recovery MRI. For all participants, volumetric MRI, DTI, and fluid‐attenuated inversion recovery sequences were assessed visually in all planes to ensure adequate coverage and to exclude artifacts, unexpected pathology, or significant motion.

### Preprocessing of Diffusion Tensor Data

Raw diffusion weighted images were affine‐aligned to the first corresponding b0 image using a linear image registration tool (FLIRT v5.5) within the FMRIB Software Library (FSL v4.1.5) [Cook et al., 2006; Smith et al., 2006]. DTI volumes were then combined for tensor fitting using Camino (http://cmic.cs.ucl.ac.uk/camino/), and FA and other DTI metrics at each voxel then derived from the tensor eigenvalues (*λ*1, *λ*2, and *λ*3; where AX = *λ*1, RD = (*λ*2 + *λ*3)/2, trace (TR) = (*λ*1 + *λ*2 + *λ*3)) and fractional anisotropy (FA) $\sqrt{\frac{3\sum_{i=1}^{3}{\left(\lambda_{i}-TR\right)}^{2}}{2\sum_{i=1}^{3}\lambda_{i}^{2}}}$. To reduce the effect of atrophy‐related distortion in registering and projecting to a common white matter skeleton and improve image alignment in creating the group‐wise skeleton a group‐wise atlas was used within the TBSS pipeline [Keihaninejad et al., 2012]. This methodology aims to improve the quality of image registration in neurodegenerative conditions by minimizing the effects of stretch and distortion seen when attempting to warp a brain affected by significant neurodegeneration onto the standard FMRIB58 image (based on 58 scans of healthy individuals aged 20–50 years). It also reduces image distortion in regions affected by severe atrophy and provides greater detail on distal parts of white matter tracts compared with the standard TBSS pipeline (compare images generated using this versus standard approaches in Supporting Information Fig. 1).

### Analysis of Diffusion Tensor Data

To assess disease effects on white matter structures a general linear model was created with disease group membership as the factor of interest and age, gender, and disease duration (as a surrogate of disease severity and stage) included as nuisance covariates. Each metric (AX/RD/TR/FA) was analyzed with the above model, and additionally with a model that included the overall mean of the metric under study as an additional covariate. The latter model reveals areas that are specifically affected after adjusting for widespread global differences, analogous to controlling for total grey matter volume in voxel‐based morphometry [Mechelli et al., 2005]. Models were contrasted with both healthy and disease control groups. A genetic subgroup analysis was performed comparing *MAPT* and *C9ORF72* mutation groups with healthy individuals, the AD disease group and with each other using the same model design. Statistical analysis was implemented using the permutation‐based (nonparametric) “*randomise*” tool within FSL with 10,000 permutations for each test. A significance threshold (*P* < 0.05) was applied after correction for multiple comparisons using family‐wise error (FWE) correction with threshold‐free cluster enhancement (TFCE) [Smith and Nichols, 2009]. Significant results were projected onto a study‐specific average brain registered to standard (Montreal Neurological Institute; MNI) space. To provide accurate anatomic localisation a series of tract‐specific masks were applied to the significant whole brain results. Tracts corresponding to major white matter structures were selected from a probabilistic atlas [Mori et al., 2004], and to adjust for the anatomical variability of the tract thresholded at a likelihood of overlap across individuals of 20%, this moderately stringent threshold is also likely to reduce contamination within regions‐of‐interest by reducing partial volume effects and erroneous inclusion of grey matter. This figure was deemed acceptable as it allowed for maximal tract coverage whilst minimising the possibility of overlap between neighbouring tracts (i.e. inferior longitudinal fasciculus and uncinate fasciculus, see Supporting Information Fig. 2). The aim was to include the core of the tract, whilst excluding peripheral parts of the tract with high inter‐individual variability [Hua et al., 2008]. A total of 14 binary masks were created, covering major white matter pathways including right and left inferior longitudinal fasciculus (ILF), superior longitudinal fasciculus (SLF), uncinate fasciculus, anterior thalamic radiation (ATR), cingulum bundle, corticospinal tract (CST), corpus callosum, and fornix. Further information on the significant (FWE‐corrected) results was extracted from within each anatomical mask (using FSL's “*fslstats*”). The proportion of significantly affected voxels within each tract was calculated by dividing the number of significant voxels identified by the total number of voxels within each tract limited to within the skeleton. In addition, to assess the anatomical reliability of results obtained from the group skeleton, result maps were de‐projected and overlaid on each individual participant's FA image, which confirmed good anatomical alignment of the results with white matter tracts.

### Sensitivity and Specificity of Diffusion Metrics in Predicting Group Membership

To determine the sensitivity and specificity of different diffusivity metrics in classifying individual participants into separate groups (bvFTD/healthy individual/AD) receiver operating characteristic (ROC) curves were constructed and areas‐under‐curve (AUC) were calculated. Two approaches were used for the classification, assessing respectively the mean of each individual metric (FA, AX, RD, TR) across the whole white matter skeleton and the mean values within individual tracts identified as prominently involved in the initial DTI analysis. Mean values were extracted using the “*fslmeants*” command. ROC curves and AUC values were calculated for both global and individual tract diffusivity data.

### Grey Matter Analysis

In order to compare white with grey matter changes in the bvFTD cohort, profiles of grey matter atrophy were first derived using voxel‐based morphometry (VBM) with the New Segment [Weiskopf et al., 2011] and DARTEL [Ashburner, 2007] toolboxes of SPM8 (http://www.fil.ion.ucl.ac.uk/spm) under Matlab 7.14 (http://www.mathworks.com). Segmentation, normalization, and modulation were performed using default parameter settings. Smoothing of grey matter images used a 6 mm kernel size. Final DARTEL transformations were combined with affine transformations to MNI space. To maintain consistency with the white matter analysis, grey matter was assessed using the same statistical methodology. Nonparametric permutation testing (*n* = 10,000) was used to examine differences in regional grey matter volume between groups and included age, gender, total intracranial volume and disease duration as nuisance covariates. Maps of grey matter atrophy were overlaid onto an MNI152 standard brain after FWE correction (*P* < 0.05) with TFCE. To provide information on the anatomical relationship of the grey and white matter changes, an intersection map of all four DTI metrics (derived by combining the individual mean‐adjusted bvFTD versus healthy control result maps, thresholded using FWE‐correction *P* < 0.05) were overlaid on the map of grey matter atrophy.

## RESULTS

### Participant Clinical Characteristics

Of the 27 patients with bvFTD identified (mean age 62.5 ± 9.0 years; male = 20) 14 met criteria for a diagnosis of definite bvFTD and 13 met criteria for a diagnosis of probable bvFTD [Rascovsky et al., 2011]. Of those with definite bvFTD nine had mutations in the *MAPT* gene (five harbored the intron 10 + 16 mutation, two harbored the exon 13 R406W mutation, one harbored the exon 10 P301S mutation, and one harbored a novel mutation in exon 12 resulting in a single amino acid substitution at codon 351 (Q351R)), four had mutations in *C9ORF72* and one patient who subsequently died was found to have Pick's disease pathology at postmortem. In addition to meeting diagnostic criteria for bvFTD, two patients had symptoms compatible with Progressive Supranucelar Palsy (both having sporadic bvFTD), two had symptoms compatible with motor neuron disease (one having a *C9ORF72* mutation, one being sporadic) and one had symptoms compatible with corticobasal syndrome (being sporadic). All AD patients had a typical memory‐led presentation. Demographic, clinical and neuropsychological data for all participant groups are summarised in Table 2. The healthy control and AD groups were comparable in age and gender characteristics (healthy control group mean age 64.5 ± 4.5 years, male = 13/20; AD group mean age 63.1 ±5.2 years; male = 17/25). CSF data was available on 13 of 27 bvFTD patients, all had profiles compatible with non‐Alzheimer's pathology (normal Abeta1–42 and normal or elevated total tau), although one subject had low Abeta1–42 and low/normal tau which was felt to be consistent with a handling error. CSF data was available on 11 of 25 AD patients, all had profiles compatible with AD pathology (low Abeta1–42, elevated total tau). A detailed general neuropsychological assessment was completed in 69 of 72 participants (one patient with bvFTD and two with AD were unable to comply with testing). Profiles of performance in the patient groups were in keeping with clinical syndromes: both disease groups showed widespread deficits compared with the healthy control group, however, the AD group performed inferiorly to the bvFTD group on tests of arithmetic and general executive capacity while the bvFTD group performed inferiorly to the AD group on semantic processing.

**Table 2:** Summary of demographic, clinical and neuropsychological data for all groups

Characteristic	bvFTD; *n* = 27 (M20:F7)		AD; *n* = 25 (M17:F8)		Controls; *n* = 20 (M13:F7)		Group comparisons: *P* value		
	Mean	SD	Mean	SD	Mean	SD	bvFTD, *v* control	bvFTD, *v*, AD	AD, *v*, control
Clinical									
Age all subjects (years)	62.5	9.0	63.1	5.2	64.5	4.5	ns	ns	ns
Sporadic	64.9	8.6							
*MAPT*	58.2	9.0							
*C9ORF72*	64.1	8.7							
MMSE (/30)	24.2	5.9	20.0	5.1	29.6	0.6	**<0.001**	**<0.01**	**<0.001**
Duration all subjects (years)	6.5	4.9	5.7	3.4	—	—	—	ns	—
Sporadic	6.0	4.4							
*MAPT*	6.0	5.4							
*C9ORF72*	9.4	5.9							
CSF (bvFTD *n* = 13, AD *n* = 11)									
Total Tau (pg/mL)	287	141	833	229					
Abeta1‐42 (pg/mL)	676	281	240	73					
Neuropsychological^a^									
VIQ	81.2	23.4	90.6	19.4	123.3	9.6	**<0.001**	ns	**<0.001**
PIQ	88.6	19.1	83.3	18.7	118.1	10.9	**<0.001**	ns	**<0.001**
Recognition memory: words (/50)	33.3	10.1	29.4	7.8	48.1	2.3	**<0.001**	0.08	**<0.001**
Recognition memory : faces (/50)	32.8	6.8	33.7	6.3	41.9	4.7	**<0.001**	ns	**<0.001**
Digit span: forward (/12)	7.1	2.2	6.3	2.2	9.1	1.4	**0.002**	ns	**<0.001**
Digit span: reverse (/12)	5.5	2.7	4.3	3.0	6.8	1.8	ns	0.09	**0.002**
BPVS (/150)	116.5	38.0	132.5	24.3	147.9	1.5	**<0.001**	**0.02**	**<0.001**
Graded naming test (/30)	10.8	9.1	13.9	8.4	26.0	2.1	**<0.001**	ns	**<0.001**
Graded arithmetic test (/24)	12.1	6.9	5.3	4.0	14.1	5.4	0.4	**<0.001**	**<0.001**
VOSP (/20)	15.7	3.2	15.0	3.3	18.8	1.1	**<0.001**	ns	**<0.001**
DKEFS: color naming (max 90 secs)	45.6	22.9	55.0	18.7	31.2	4.7	**0.03**	**0.03**	**<0.001**
DKEFS: word naming (max 90 secs)	35.1	23.6	36.6	15.3	21.5	3.4	**0.02**	0.1	**<0.001**
DKEFS: ink naming (max 180 secs)	100.4	48.4	141.0	44.0	56.8	11.5	**<0.001**	**0.003**	**<0.001**

Group comparisons significant at *P* < 0.05 are shown in bold.

a Neuropsychological data not collected for one patient with bvFTD and two patients with AD.

AD, Alzheimer's disease; BPVS, British Picture Vocabulary Scale; DKEFS, Delis‐Kaplan Executive Function System; F, female; bvFTD, frontotemporal dementia; M, male; MMSE, Mini‐Mental State Examination score; ns, non‐significant. PIQ, performance IQ; SD, standard deviation (values shown in italics); VIQ, verbal IQ; VOSP, Visual Object and Space Perception battery. See Supporting Information for details of neuropsychological tests used.

### White Matter Tract Alterations in Frontotemporal Dementia

**Figure 1:**
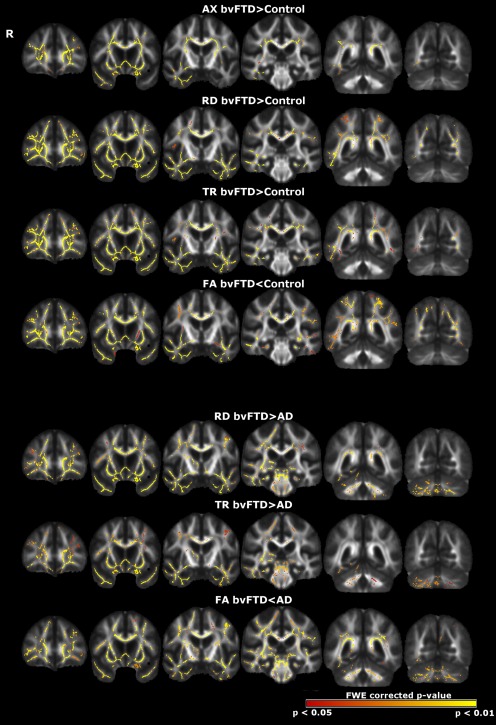
Unadjusted white matter tract data: Patterns of white matter alteration in the behavioral variant frontotemporal dementia (bvFTD) group compared with the healthy control group (above, rendered on representative coronal MRI sections from a customised group template and displayed in standard space; the right hemisphere (R) is shown on the left) and the Alzheimer's disease (AD) group (below, rendered on representative coronal sections), uncorrected for global diffusivity effects. Results for particular diffusivity metrics (AX, axial diffusivity; FA, fractional anisotropy; RD, radial diffusivity; TR, trace diffusivity) are shown separately. The colour scale indexes *P* values after family‐wise error correction for multiple comparisons over the whole brain. [Color figure can be viewed in the online issue, which is available at http://wileyonlinelibrary.com.]

**Figure 2:**
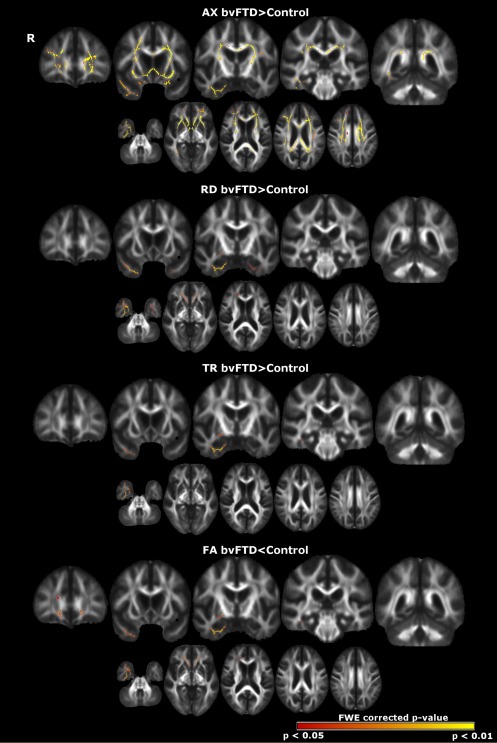
Adjusted white matter tract data: Patterns of white matter alteration in the behavioral variant frontotemporal dementia (bvFTD) group compared with the healthy control group (rendered on representative coronal and axial MRI sections from a customised group template and displayed in standard space; the right hemisphere (R) is shown on the left), after correction for mean global diffusivity value. Results for particular diffusivity metrics (AX, axial diffusivity; FA, fractional anisotropy; RD, radial diffusivity; TR, trace diffusivity) are shown separately. The colour scale indexes *P* values after family‐wise error correction for multiple comparisons over the whole brain. [Color figure can be viewed in the online issue, which is available at http://wileyonlinelibrary.com.]

**Figure 3:**
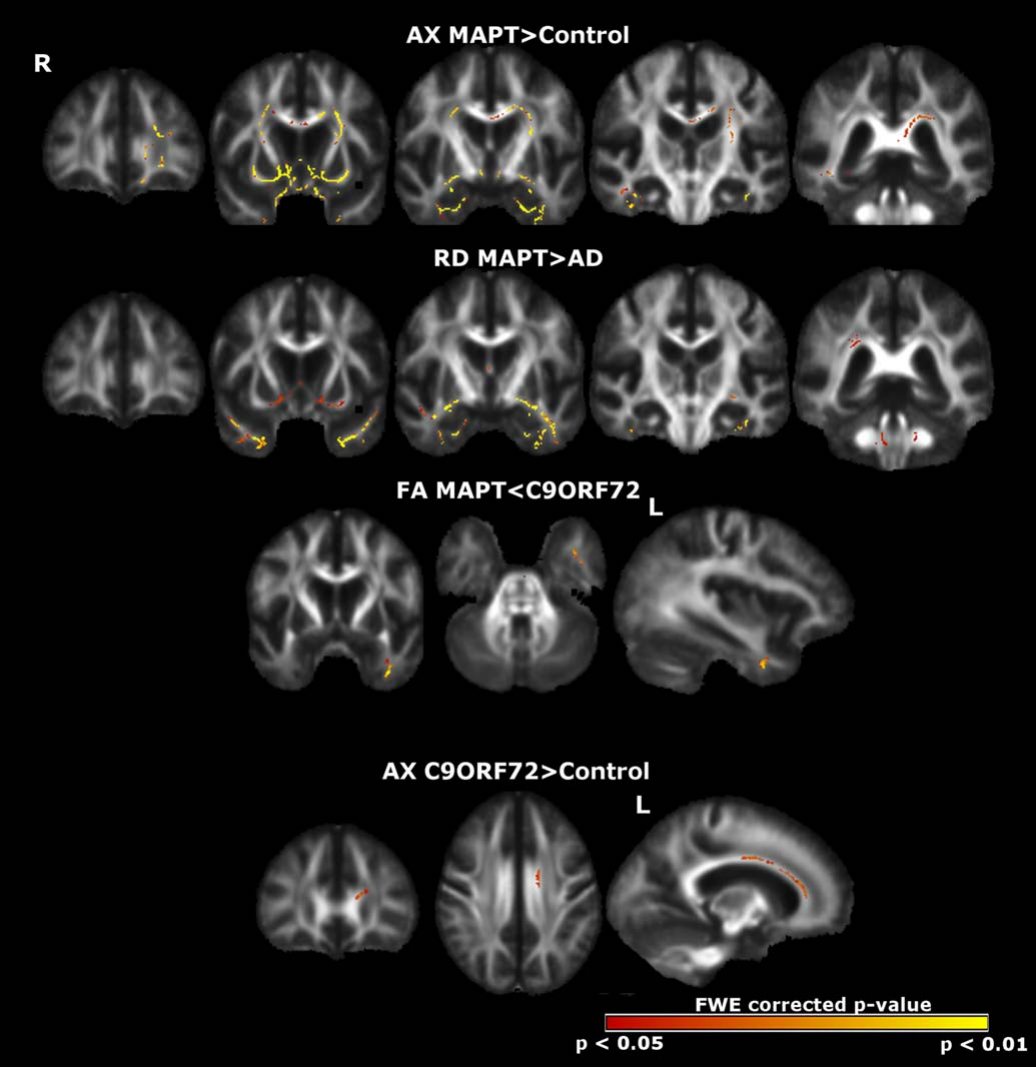
Adjusted white matter tract data: Patterns of white matter alteration in the *MAPT* and *C9ORF72* mutation groups compared with the healthy control group and the Alzheimer's disease (AD) group (rendered on representative coronal, axial and sagittal MRI sections from a customised group template and displayed in standard space; the right hemisphere (R) is shown on the left; sagittal sections display left hemisphere (L)), after correction for mean global diffusivity value. Results for AX (axial diffusivity), RD (radial diffusivity) and FA (fractional anisotrophy) are thresholded at *P* < 0.05 FWE correction. The colour scale indexes *P* values after family‐wise error correction for multiple comparisons over the whole brain. [Color figure can be viewed in the online issue, which is available at http://wileyonlinelibrary.com.]

**Figure 4:**
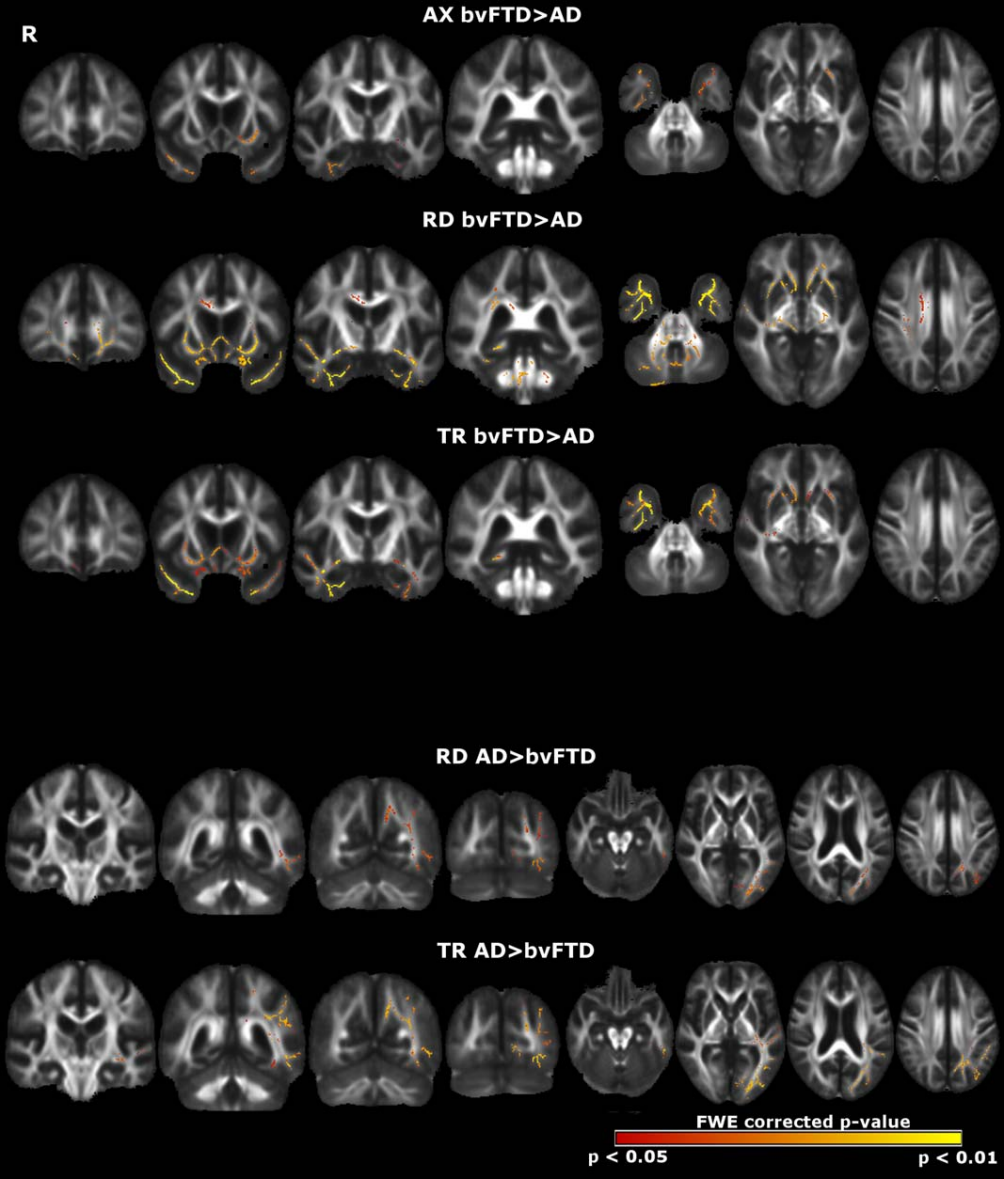
Adjusted white matter tract data: Patterns of white matter alteration in the behavioral variant frontotemporal dementia (bvFTD) group compared with the Alzheimer's disease (AD) group (rendered on representative coronal and axial MRI sections from a customised group template and displayed in standard space; the right hemisphere (R) is shown on the left), after correction for mean global diffusivity value. Results for particular diffusivity metrics (AX, axial diffusivity; FA, fractional anisotropy; RD, radial diffusivity; TR, trace diffusivity) are shown separately. Panels above show white matter damage greater in bvFTD than AD, panels below show white matter damage greater in AD than bvFTD. The colour scale indexes *P* values after family‐wise error correction for multiple comparisons over the whole brain. [Color figure can be viewed in the online issue, which is available at http://wileyonlinelibrary.com.]

Widespread white matter tract pathology was identified in the bvFTD group compared with both the healthy control group and the AD group (Figs. 1, 2, 3, 4). Data quantifying the extent and significance of alteration within the most affected white matter tracts compared with healthy controls and AD are listed in Tables 3 and 4, respectively; complete data on each metric and region of interest are provided in Supporting Information (Supporting Information Tables I to III). Here we first report data without correction for global DTI metric effects, in order to present a complete picture of white matter tract alterations; we then report data after global DTI metric correction, identifying white matter pathology with greater anatomical specificity.

**Table 3:** Unadjusted diffusion data by metric and region of interest

bvFTD versus healthy control group								
Tract	Radial diffusivity		Trace diffusivity		Axial diffusivity		Fractional anisotropy	
	*P* value	% Extent	*P* value	% Extent	*P* value	% Extent	*P* value	% Extent
Uncinate fasciculus: right	0.001	99.5	0.002	99.2	0.004	68.6	0.003	89.0
Unicinate fasciculus: left	0.002	95.3	0.002	92.5	0.002	62.2	0.003	76.5
Cingulum bundle: right	0.003	82.5	0.01	66.1	0.01	24.6	0.007	83.1
Cingulum bundle: left	0.004	74.8	0.007	61.9	0.009	16.6	0.004	78.9
Corpus callosum	0.004	65.2	0.005	66.0	0.006	45.1	0.005	63.6

Only region of interests where at least one metric had greater than 50% extent are displayed. % extent is the percentage of significant voxels divided by total number of voxels within the masked region of the skeleton. Complete data on each metric and region of interest are provided in Supporting Information.

**Table 4:** Unadjusted diffusion data by metric and region of interest

bvFTD group versus Alzheimer's disease group						
Tract	Radial diffusivity		Trace diffusivity		Fractional anisotropy	
	*P* value	% Extent	*P* value	% Extent	*P* value	% Extent
Uncinate fasciculus: right	0.002	94.4	0.006	86.2	0.004	89.3
Unicinate fasciculus: left	0.003	89.9	0.006	84.1	0.004	86.1
Cingulum bundle: left	0.003	61.8	0.008	49.2	0.004	69.0
Corpus callosum	0.007	51.0	0.008	40.4	0.006	59.8

Regions of interest where at least one metric had greater than 50% extent are displayed. % extent is the percentage of significant voxels divided by total number of voxels within the masked region of the skeleton. Complete data on each metric and region of interest are provided in Supporting Information.

Compared with the healthy control group (Table 3 and Supporting Information Table I), the bvFTD group showed most significant and consistent white matter pathology across diffusivity metrics (increased AX/RD/TR, decreased FA in bvFTD) in bilateral uncinate fasciculus, cingulum bundle, corpus callosum, and somewhat less prominently in SLF, ILF, ATR, and fornix. Comparing DTI metrics, the most extensive white matter damage (% of significant voxels within a white matter tract) was detected in right uncinate fasciculus using TR and RD. Compared with the AD group (Table 4 and Supporting Information Table II), the bvFTD group again showed most significant and consistent white matter pathology across metrics (increased RD/TR, decreased FA in bvFTD) in bilateral uncinate fasciculus, cingulum bundle, and corpus callosum, and somewhat less prominently in SLF, ILF, ATR, and fornix; comparing DTI metrics, comparably extensive white matter damage was detected in right uncinate fasciculus using RD, FA or TR. The reverse contrasts (increased AX/TR/RD, decreased FA in the healthy control and AD groups) did not identify any areas of significant white matter alteration.

After correction for the effects of global mean diffusivity values, more focal profiles of white matter alteration emerged. Compared with the healthy control group (Supporting Information Table I see also DTI intersection map in Fig. 6), the bvFTD group showed most significant and consistent white matter tract pathology across metrics (increased AX/TR/RD, decreased FA in bvFTD) in right uncinate fasciculus; comparing DTI metrics, the most extensive white matter damage was detected in right uncinate fasciculus using AX. Compared with the AD group (Supporting Information Table II), the bvFTD group showed most significant and consistent white matter pathology across metrics (increased AX/TR/RD in bvFTD) in bilateral uncinate fasciculus; comparing DTI metrics, the most extensive white matter damage was detected in right uncinate fasciculus using RD followed by TR. Conversely, compared with the bvFTD group, the AD group showed greater white matter damage (increased TR/RD in AD) in posterior left hemispheric white matter tracts (SLF and ILF); comparing DTI metrics, the most extensive white matter alterations in the AD group compared with bvFTD group were detected in left ILF using TR.

**Figure 5:**
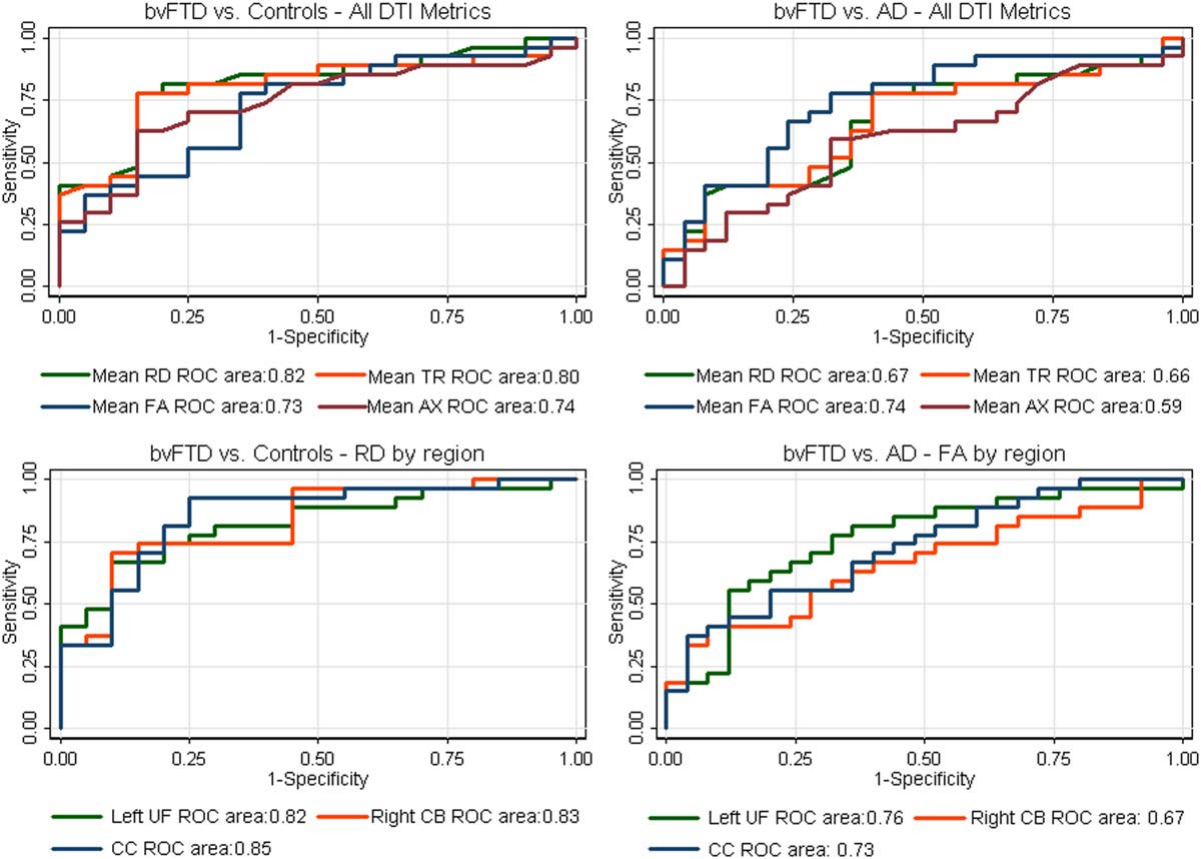
Receiver operating characteristic (ROC) curves for classification of individuals to the behavioral variant frontotemporal dementia (bvFTD) group versus the healthy control group (left) and Alzheimer's disease (AD) group (right), based on each participant's global mean diffusivity data (above) and each participant's unadjusted mean diffusivity data within a tract region of interest (below). ROC curves for particular diffusion tensor imaging (DTI) metrics (AX, axial diffusivity; FA, fractional anisotropy; RD, radial diffusivity; TR, trace diffusivity) and white matter tracts of interest (CB, cingulum bundle; CC, corpus callosum; UF, uncinate fasciculus) are shown separately; area‐under‐curve is displayed for each curve (area‐under‐curve = 1 corresponds to ideal separation of groups). [Color figure can be viewed in the online issue, which is available at http://wileyonlinelibrary.com.]

**Figure 6:**
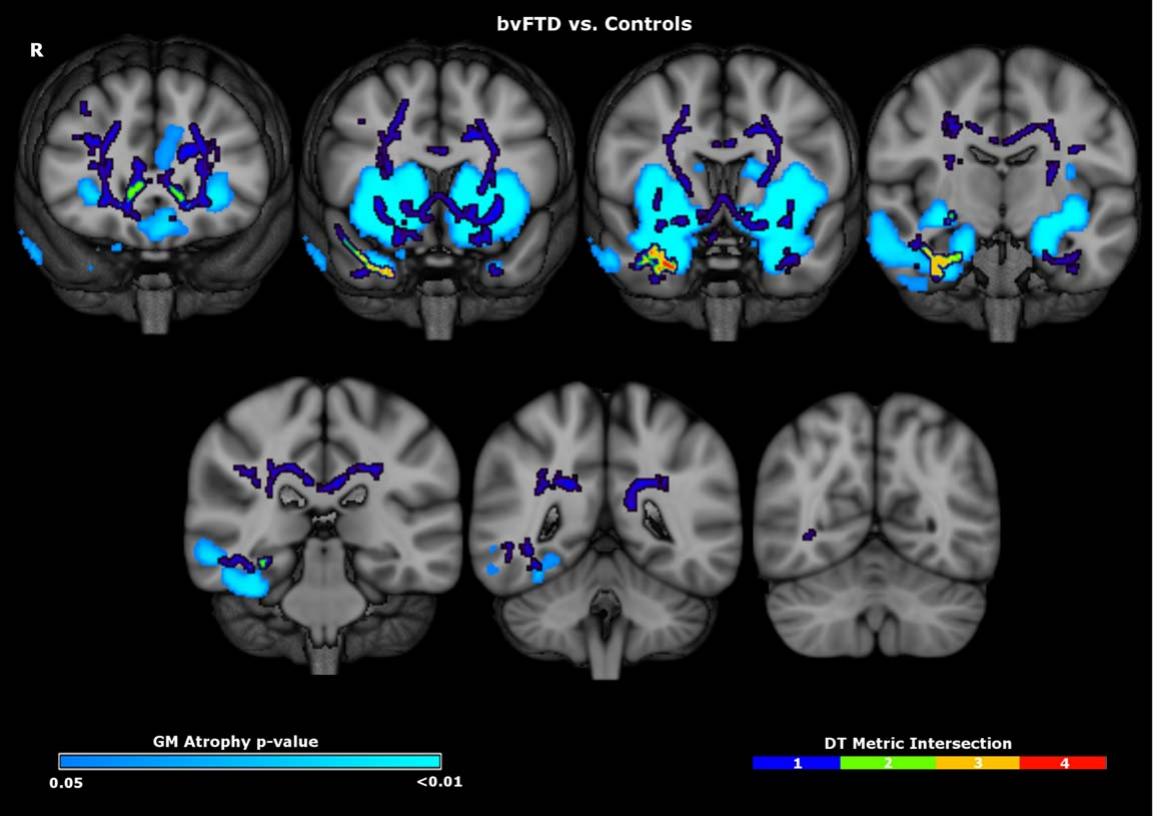
Maps of grey matter (GM) atrophy (light blue) and regions of intersection of all four white matter diffusivity metrics (adjusted for mean global diffusivity value) in the behavioral variant frontotemporal dementia (bvFTD) group compared with the healthy control group. Maps are overlaid on representative coronal sections of the MNI152 template brain; the right hemisphere (R) is shown on the left. The diffusivity tensor (DT) metric data intersection colour scale indexes the extent of overlap between metrics, i.e. dark blue indicates change in only one metric whereas red indicates change in all four metrics; the GM colour scale indexes *P* values after family‐wise error correction for multiple comparisons over the whole brain. [Color figure can be viewed in the online issue, which is available at http://wileyonlinelibrary.com.]

### White Matter Tract Alterations in Genetic Subgroups

Analyses based on small numbers of cases revealed white matter tract signatures associated with particular genetic subgroups of bvFTD (mutations in *MAPT* vs. *C9ORF72*) after global mean value correction (Fig. 3 and Supporting Information Table III). Compared to the healthy control group, the *MAPT* mutation subgroup showed consistent alterations in left uncinate fasciculus across diffusivity metrics, albeit highly variable in extent within the tract; the most extensive alterations were detected with AX, which revealed additional involvement of right uncinate fasciculus, corpus callosum, fornix and bilateral ILF and SLF. Compared with the AD group, the *MAPT* mutation subgroup showed altered RD and TR in CST, ATR, ILF and uncinate fasciculus; again, these alterations varied widely in extent between diffusivity metrics. Alterations detected in the smaller *C9ORF72* mutation subgroup were less extensive: compared with the healthy control group, the *C9ORF72* mutation cases showed increased AX in corpus callosum and cingulum bundle. On direct comparison of *MAPT* with *C9ORF72* those with *MAPT* showed alterations in white matter within the left anterior temporal pole, as measured by reduced FA. Contrasts of *MAPT* and *C9ORF72* mutations subgroups with sporadic bvFTD were examined but did not reach statistical significance.

### Comparison of Diffusivity Metrics

Comparing AUC data between DTI metrics (Fig. 5), whole brain mean RD had the greatest sensitivity (82%) and specificity (80%) for distinguishing the bvFTD group from the healthy control group; TR performed comparably, and AX and FA somewhat less favourably. Comparing specific white matter tracts of interest using mean RD (Fig. 5), corpus callosum, left uncinate fasciculus, and left cingulum bundle showed high sensitivity (93%, 82%, and 74%, respectively) and specificity (75%, 75%, and 70%, respectively) for distinguishing the bvFTD group from the healthy control group. Whole brain mean FA had the greatest sensitivity (78%) and specificity (68%) for distinguishing the bvFTD group from the AD group (Fig. 5); RD, TR, and AX performed somewhat less favourably. Comparing specific white matter tracts of interest using mean FA (Fig. 5), left uncinate fasciculus showed the greatest sensitivity (77%) and specificity (68%); left cingulum bundle and corpus callosum showed slightly lower sensitivity (63% and 56%, respectively) but greater specificity (80% for both).

### White Matter Tract Alterations Compared With Grey Matter Atrophy

In the VBM analysis, compared with the healthy control group the bvFTD group showed, as anticipated, areas of significantly (*P* < 0.05 after whole‐brain FWE correction for multiple voxel‐wise comparisons with TFCE) reduced grey matter predominantly distributed in frontal and temporal cortices in both cerebral hemispheres (Fig. 6). Regions of grey matter atrophy included bilateral orbitofrontal cortex, superior and inferior frontal gyri, insular cortex, left cingulate gyrus, bilateral amygdala, right middle, and inferior temporal gyri. Compared with the healthy control group the AD group also showed the anticipated profile of grey matter atrophy, predominantly involving medial temporal lobe and temporo‐parietal regions (Supporting Information Fig. 3). The intersection map of DTI metrics with grey matter atrophy in the bvFTD group (Fig. 6) showed that white matter damage generally occurred in close anatomical proximity to areas of grey matter atrophy, particularly in right anterior temporal lobe and for uncinate fasciculus. However, compared with the distribution of grey matter atrophy, white matter damage across diffusivity metrics was spatially more extensive, particularly dorsally and posteriorly within the hemispheres. White matter changes (assessed using AX or FA) also extended beyond apparent zones of grey matter atrophy in the AD group (Supporting Information Fig. 3).

## DISCUSSION

Here we have employed quantitative, image registration‐based DTI methods to demonstrate profiles of white matter tract damage in a large cohort of patients with bvFTD, representing the major genetic and sporadic disease subtypes. Considering the bvFTD cohort firstly as a whole, white matter changes in comparison to healthy older individuals were extensive, distributed in both cerebral hemispheres with particularly prominent involvement of the uncinate fasciculus, cingulum bundle and corpus callosum. This white matter profile was identified after taking disease severity into account; moreover, a similar emphasis of white matter tract alterations was identified when the bvFTD group was compared with the AD group, suggesting that involvement of these tracts may be a relatively specific index of pathologies in the bvFTD spectrum. The findings were further consolidated by adjusting for the effects of global mean diffusivity: after global adjustment, involvement of uncinate fasciculus was a prominent and consistent indicator of bvFTD pathology compared both with healthy individuals and patients with AD.

These findings build upon and extend previous studies of white matter tract involvement in bvFTD [Agosta et al., 2011; Borroni et al., 2007; Matsuo et al., 2008; Whitwell et al., 2010; Zhang et al., 2011, 2009, 2013]. Comparing tract profiles in the present study with previous work, the majority of studies have shown involvement of uncinate facsciculus, corpus callosum, ILF, and cingulum bundle. However, the extent of reported white matter pathway involvement has varied considerably between studies. The extensive profile of white matter alterations we have demonstrated here may be in part attributable to the use of a customised white matter template as well as the comparatively large cohort, which is likely to have sampled a range of underlying pathologies within the bvFTD spectrum, as evidenced by both the genetic data and the presence of clinical features, in some participants, compatible with particular underlying pathologies.

The white matter pathways delineated here are plausible candidates to mediate brain network dysfunction and the most prominently involved tracts have previously been correlated with a range of cognitive deficits in the bvFTD syndrome [Tartaglia et al., 2012]. The uncinate fasciculus has been associated with a range of cognitive functions and recently has been particularly implicated in the evaluation of affective and other inter‐personal signals [Von Der Heide et al., 2013], and in the pathogenesis of neuropsychiatric symptoms [Phan et al., 2009] and risk‐taking behaviors [Linke et al., 2013]: such processes are likely to be relevant to various canonical features of bvFTD [Rascovsky et al., 2011]. The uncinate fasciculus has also been directly implicated in the modulation of inhibition in patients with bvFTD [Hornberger et al., 2011]. The cingulum bundle is intimately associated with cingulate cortex, previously identified as a key component of the ‘salience network’ implicated in the pathogenesis of the bvFTD syndrome [Kim et al., 2012; Seeley et al., 2009; Zhou et al., 2012]. Both cingulum and corpus callosum have been implicated in the pathogenesis of obsessive–compulsive symptoms and executive dysfunction [Bora et al., 2011; Linke et al., 2013]. The ATR has been linked to aspects of attention and executive function [Andreasen et al., 1996; Schmahmann and Pandya, 2008; Van der Werf et al., 2003] as well as the pathogenesis of autism [Cheon et al., 2011]; while the fornix has recently been identified as a key locus of damage in bvFTD [Hornberger et al., 2012]. The disease specificity of other white matter associations identified here, in particular, involvement of the long intra‐hemispheric tracts SLF and ILF, is less clear: portions of these tracts were found to be involved more prominently in either bvFTD or AD. This apparent paradox may reflect evolution of pathology across white matter pathways common to the distributed brain networks that are targeted in these different neurodegenerative diseases. However, longitudinal data comparing syndromes and diseases will be required to resolve this.

The present work further suggests that DTI can delineate molecularly defined profiles of white matter pathology within the broader bvFTD spectrum. Most striking were alterations within the uncinate fasciculus in those with *MAPT* mutations, which persisted when compared with AD and *C9ORF72* groups. These more localized changes suggest that *MAPT* mutations may direct pathological changes to target this ventromedial network. In contrast those with *C9ORF72* mutations tended to have more dorsal white matter tract pathology, which targeted the cingulum bundle and corpus callosum. These findings are in line with evidence from previous neuroanatomical studies of genetic subtypes of bvFTD [Mahoney et al., 2012a, 2012b; Rohrer et al., 2011]. Notably we did not generate a significant result when *MAPT* was contrasted with the sporadic bvFTD group. The absence of a result may suggest that those with sporadic bvFTD and *MAPT* mutations have overlapping profiles of white matter pathology, at least in part, which is supported by the prominence of uncinate fasciculus involvement seen when considering all bvFTD patients collectively. This subgroup analysis does not fully resolve neuroanatomical subtyping of molecularly defined bvFTD: case numbers across subgroups were small (precluding, for example, differentiation of individual mutations within each subgroup) while certain key bvFTD phenotypes (in particular, bvFTD due to progranulin gene mutations and sporadic forms of bvFTD associated syndromes of atypical parkinsonism or motor neurone disease) were either underrepresented or not represented at all. The likely variability of pathologies within the sporadic bvFTD group may also have limited the ability to detect white matter changes specific to this group. Taking these caveats into account, the present findings provide further evidence that the profile of regional brain network disintegration in bvFTD may be modulated by underlying molecular pathologies [Warren et al., 2012] and that DTI can assist in differentiating these pathologies in vivo.

The compatibility of different diffusivity metrics is an issue of considerable clinical and neurobiological relevance to the application of DTI in neurodegenerative disease. While our findings suggest partial convergence of metrics for particular white matter tracts, we found additional substantial divergence in the white matter profiles generated using different diffusivity metrics across both sporadic and genetic forms of bvFTD (and indeed, also within the AD neurodegenerative control group; compare Figures 1 to 4 and 6, and Supporting Information Tables I to III). In principle, there is likely to be a trade‐off between higher sensitivity and higher specificity for any candidate DTI metric, presenting a practical problem of optimisation if particular metrics are in future to be used as disease biomarkers. It may be that some DTI metrics have higher inter‐subject variability, in both healthy and affected individuals, and after discounting for these affects (as we do by adjusting for global mean diffusivity), the overall extent of change may be less emphatic. After adjusting for global mean diffusivity values to enhance regional anatomical specificity, we identified disproportionate involvement of uncinate fasciculus, suggesting it may be a candidate for monitoring white matter changes in bvFTD across diffusivity metrics. This was further corroborated by a formal ROC analysis assessing the separability of bvFTD from healthy individuals and patients with AD (Fig. 5). Comparing global brain diffusivity values, RD and TR appeared most sensitive in distinguishing bvFTD from healthy controls, in keeping with previous work [Acosta‐Cabronero et al., 2010; Agosta et al., 2011; Mahoney et al., 2013] and suggesting these metrics might potentially assist in early bvFTD diagnosis. On the other hand, FA appeared to be have a greater specificity in distinguishing bvFTD from AD, suggesting that this metric might be preferred as a future disease biomarker (for example, in treatment trials targeting particular pathologies). However, data in the genetic subgroups (see Supporting Information Table III) suggest that the “optimal” diffusivity metric may be further stratified within the bvFTD spectrum. These discrepant findings between DTI metrics may have face validity. Firstly, whilst previous studies suggest that AX or RD may be superior to FA in detecting white matter pathology in neurodegeneration, none have made direct comparisons between different disease groups. Secondly, a number of studies within other degenerative conditions have demonstrated that FA has some specificity in distinguishing different disease subtypes[Menke et al., 2012; Prakash et al., 2009] This underlines the need to take the specific application into account when evaluating candidate DTI metrics [Acosta‐Cabronero et al., 2010]. Variation among DTI metrics may in addition potentially hold neurobiological insights: for example, the apparent discrepancy between RD and FA in signifying bvFTD (whether referenced to healthy controls or to AD) might be driven by changes in either RD or AX as change in either metric could affect FA.

A related key issue concerns the relationship of grey matter atrophy to white matter damage in bvFTD. Here, changes in white matter tracts occurred in close anatomical proximity to regions of grey matter loss but were more spatially distributed (see Fig. 6). In line with this another recent study has also found somewhat more widespread white matter changes in bvFTD when compared with AD [Zhang et al., 2011]. White matter and grey matter alterations were most robustly co‐localized in the right anterior temporal lobe including the most consistently involved white matter tract, the uncinate fasciculus. There is a need for caution when comparing different neuroimaging modalities, such as DTI and VBM. However, the greater spatial extent of white matter damage might reflect distal propagation of pathology from primarily involved regions adjacent to regions of grey matter damage (due for example to pathogenic protein diffusion or withdrawal of trophic support) [Hardy and Revesz, 2012; Warren et al., 2012]; alternatively, however, white matter pathology might drive grey matter loss, such that over time grey matter atrophy spreads to become more contiguous with white matter damage. Substantial emerging histopathological evidence suggests that white matter pathology may be significant in bvFTD [Ahmed et al., 2011; Hiji et al., 2008; Neumann et al., 2007], may develop early in the disease process [Broe et al., 2004; Martin et al., 2001] and may promote subsequent upstream cortical degeneration [Drzezga et al., 2011; Villain et al., 2008]. Detailed longitudinal analyses assessing the evolution of white matter and grey matter alterations in tandem will be required to resolve these possibilities.

## CONCLUSIONS

This work has identified profiles of white matter tract damage in a large, representative cohort of patients with bvFTD, demonstrating a distributed signature of white matter alterations that is likely to be core to the pathophysiology of the syndrome and suggesting that this signature is modulated by underlying molecular pathologies. The study has highlighted a number of directions for further work. Important limitations of the present study include a sparsity of particular genetic and clinical bvFTD subtypes and lack of histopathological correlation. Analysis of altered structural connectivity may be further refined by inclusion of robust clinical severity and neuropsychological measures within future imaging datasets. Future studies will also likely require multi‐centre collaborations in order to recruit substantial case numbers across the bvFTD spectrum and among disease controls, to follow these cohorts longitudinally with white and grey matter imaging modalities, and to correlate phenotypic with pathological features. Ideally this should also include histomorphometry to assess the relationship between microstructural diffusion imaging changes, histopathological changes in WM tracts and regional grey matter damage. In addition to these logistical and neurobiological challenges, this study has identified a number of technical challenges in the application and interpretation of DTI in a heterogeneous syndrome such as DTI. Foremost amongst these is the richness of most DTI datasets: it remains unclear how best to assess diffusivity metrics in relation to one another, or whether peak change, extent of change or some combination is the optimal index of disease. This is true even for unitary pathologies such as AD but is compounded in the face of diverse histopathological substrates in bvFTD. The present data show extensive overlap between metrics and tracts among diseases (bvFTD and AD) and putative bvFTD subtypes. Ultimately, the solution may lie in the use of multivariate or multimodal indices and composite profiles of tract alteration that are informed by an improved understanding of the pathobiology of white matter diffusion and the natural history of particular bvFTD pathologies [Avants et al., 2010; Groves et al., 2011; Naylor et al., in press] A further challenge is the anatomical configuration of relevant white matter pathways in bvFTD (in particular, crossing fibres) which may only be resolved by improved imaging pipelines [Jbabdi et al., 2010] and using methods such as tractography to assess in greater detail the alterations within particular tracts. These many challenges notwithstanding, our findings together with previous work provide a clear rationale for the further evaluation of DTI as a candidate clinical biomarker and neurobiological probe in bvFTD.

## ACKNOWLEDGMENTS

The authors thank all patients and healthy volunteers for their participation. The Dementia Research Centre is an Alzheimer's Research UK Co‐ordinating Centre.

## Supporting information

Additional Supporting Information may be found in the online version of this article

Supporting Information
