# Supplementary material for: Profiles of white matter tract pathology in frontotemporal dementia
Source: Hum Brain Mapp. 2014 Feb 7;35(8):4163–79. doi: 10.1002/hbm.22468 (PMC4312919; doi:10.1002/hbm.22468)
Supplement: Supplementary file 1 — Supporting Information [file HBM-35-4163-s001.docx]

**Supplementary Material**

**Neuropsychological tests**

The Recognition Memory Test for faces (Warrington, 1984) presents 50 faces with an orienting question. The participant is then presented with the target paired with an unfamiliar face and asked to judge which they have seen before. The same procedure is used for the Recognition Memory Test for words. The British Picture Vocabulary Scale (Dunn, Dunn & Whetton, 1982) tests comprehension with a non-verbal output. Participants must match one of four pictures to a target word of increasing difficulty. The Graded Naming Test (McKenna & Warrington, 1983) requires participants to name line drawings that become increasingly less common. The Graded Difficulty Arithmetic Test (Jackson & Warrington, 1986) allows participants 10 seconds to perform increasingly difficult mental arithmetic sums, using addition and subtraction. For the Object Decision subtest of the Visual Object and Space Perception battery (Warrington & James, 1991) participants are asked to identify the silhouette of a 75 degree rotated real object from three nonsense silhouettes of similar complexity. The Delis-Kaplan Executive function system Colour-word Interference test assess executive function using a progressively challenging set of tests, first requiring subjects to name consecutive coloured rectangles, then read aloud printed colour names and finally identify the ink colour where colour words are presented in a conflicting colour ink.

*References:*

Delis, D. C., Kaplan, E., & Kramer, J. H., 2001. *Delis–Kaplan Executive Function System (D-KEFS): Examiner’s manual*. San Antonio, TX: The Psychological Corporation.

Dunn, L. M., Dunn, L. M. & Whetton, V., 1982. *British Picture Vocabulary Scale – Revised*. Windsor: UK. NFER-Nelson.

Jackson, M. & Warrington, E. K., 1986. Arithmetic skills in patients with unilateral cerebral lesions. *Cortex*, *22*, 611–620.

McKenna, P., & Warrington, E.K., 1983. *The Graded Naming Test.* Windsor, Berks: NFER-Nelson.

Warrington, E. K., & James, M., 1991. *The Visual Object and Space Perception Battery (VOSP)*. Bury St. Edmunds, England: Thames Valley Test Co.

Warrington, E. K., 1984. *Recognition Memory Test: Manual*. Berkshire UK: NFER-Nelson.


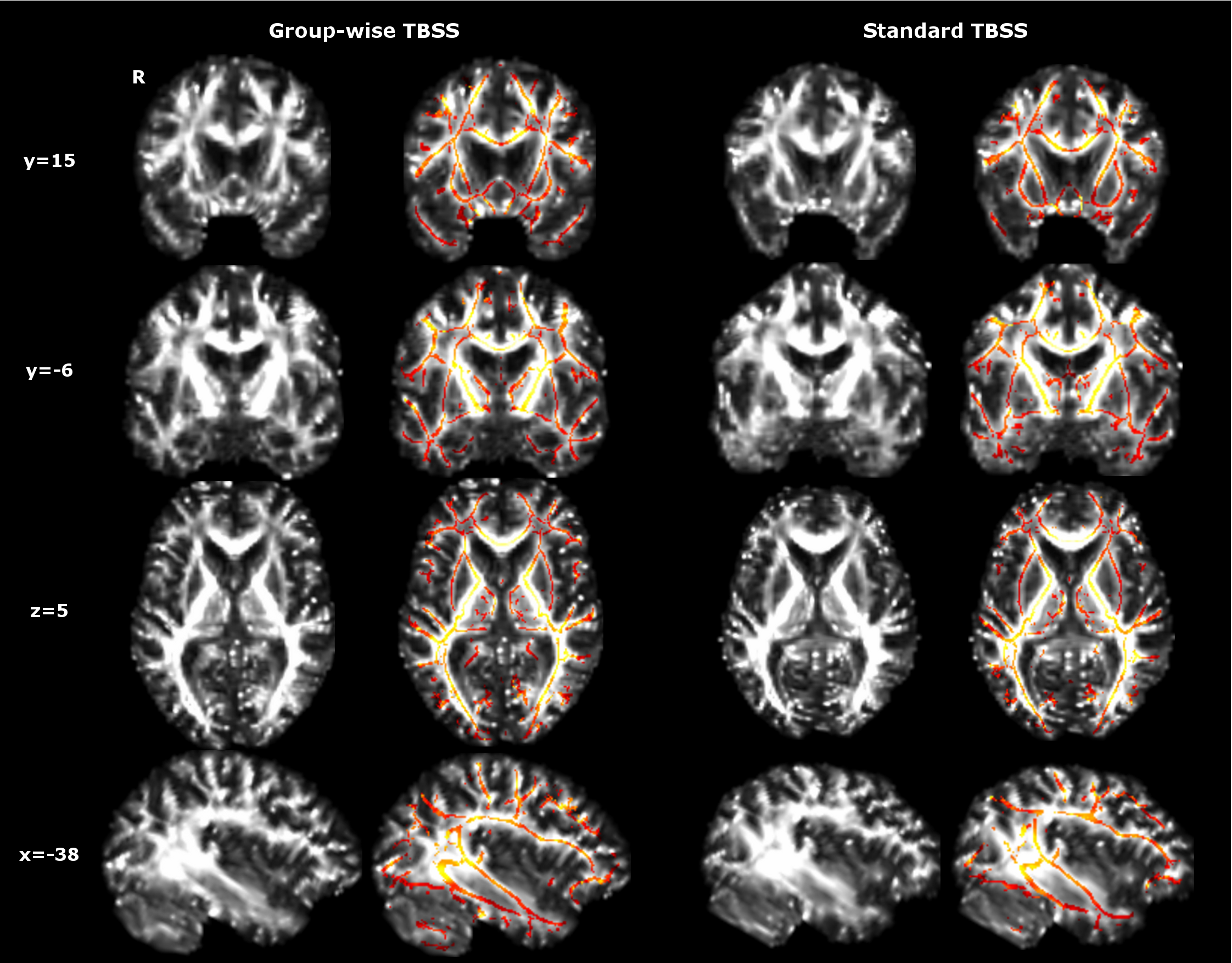


**Supplementary Figure 1** Comparison of group-wise tract-based spatial statistics (TBSS) image pipeline (left) with standard TBSS pipeline (right) performed on the same single participant. Images reflect single participant fractional anisotropy volume registered to the group-wise template on left and FMRIB58 template on right. The participant’s white matter skeleton is overlaid (red-yellow) on right of each panel. Spatial coordinates of the plane of each section are indicated on the left of the figure.


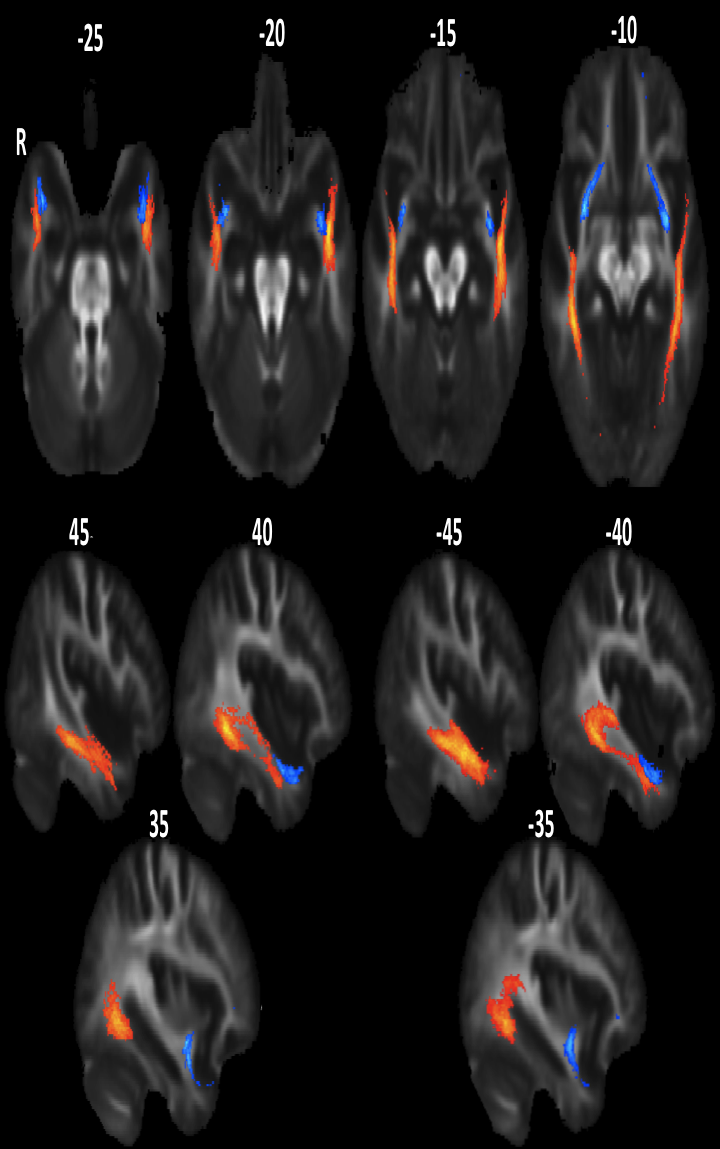


**Supplementary Figure 2.** JHU probabilistic white matter tract masks of inferior longitudinal fasciculus (red-yellow) and uncinate fasciculus (blue). Masks have been thresholded at 20% and overlaid on the study specific mean FA brain. Minimal overlap between masks is observed at this threshold. MNI coordinates of the plane of each section are indicated above each figure. R, right.


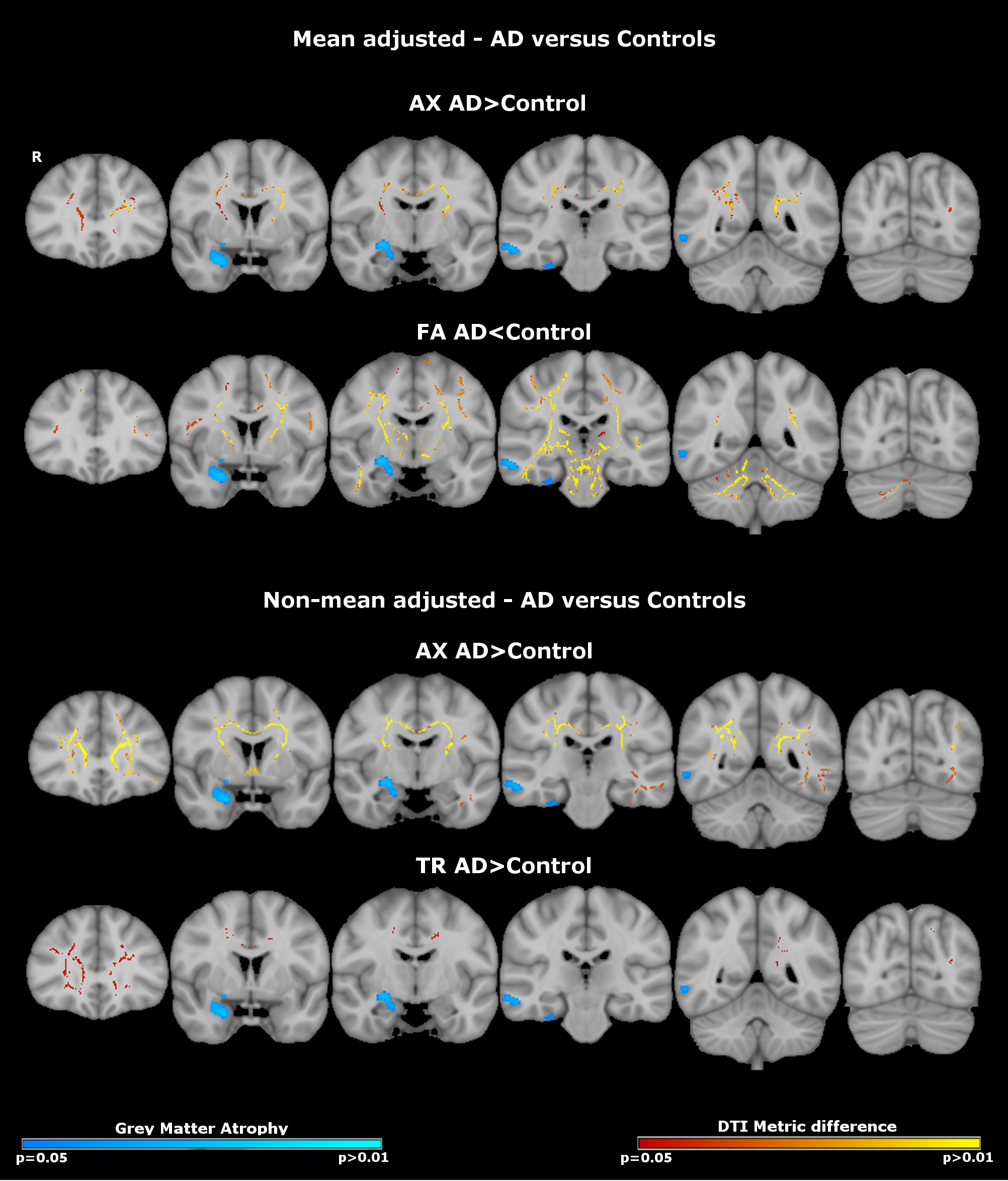


**Supplementary Figure 3.** Patterns of white matter tract alteration (red-yellow) and grey matter atrophy (blue) in the Alzheimer’s disease (AD) group compared with healthy individuals. Maps are overlaid on representative coronal sections of the MNI152 template brain; the right hemisphere is shown on the left. The top two panel displays results adjusted for mean global diffusivity, the lower two panels display unadjusted results. Key: AX, Axial diffusivity; DTI, diffusion tensor imaging; FA, Fractional Anisotropy; TR, Trace Diffusivity. Colour scales index p-values after family-wise error correction for multiple comparisons over the whole brain.

| **Unadjusted tract results** | | | | | | | | | | | | | | | |
| --- | --- | --- | --- | --- | --- | --- | --- | --- | --- | --- | --- | --- | --- | --- | --- |
| **RD bvFTD>Controls** | | | | **TR bvFTD>Controls** | | | | **AX bvFTD>Controls** | | | | **FA bvFTD<Controls** | | | |
| Tract | p-value | Voxels | % Voxels | Tract | p-value | Voxels | % Voxels | Tract | p-value | Voxels | % Voxels | Tract | p-value | Voxels | % Voxels |
| R UF | 0.001 | 390 | 99.5 | R UF | 0.002 | 389 | 99.2 | R UF | 0.004 | 269 | 68.6 | R UF | 0.003 | 349 | 89.0 |
| L UF | 0.002 | 623 | 95.3 | L UF | 0.002 | 605 | 92.5 | L UF | 0.002 | 407 | 62.2 | R CB | 0.007 | 152 | 83.1 |
| R CB | 0.003 | 151 | 82.5 | R CB | 0.01 | 121 | 66.1 | CC | 0.006 | 6717 | 45.1 | L CB | 0.004 | 669 | 78.9 |
| L CB | 0.004 | 634 | 74.8 | CC | 0.005 | 9818 | 66.0 | R ILF | 0.01 | 580 | 31.1 | L UF | 0.003 | 500 | 76.5 |
| CC | 0.004 | 9700 | 65.2 | L CB | 0.007 | 525 | 61.9 | R SLF | 0.008 | 598 | 25.1 | CC | 0.005 | 9470 | 63.6 |
| L ILF | 0.01 | 1069 | 40.6 | L ILF | 0.01 | 1093 | 41.5 | R CB | 0.01 | 45 | 24.6 | R SLF | 0.03 | 893 | 37.5 |
| R SLF | 0.02 | 899 | 37.8 | R ILF | 0.02 | 750 | 40.3 | R ATR | 0.007 | 271 | 21.9 | L ILF | 0.01 | 875 | 33.2 |
| R ILF | 0.007 | 542 | 29.1 | R SLF | 0.01 | 845 | 35.5 | L ATR | 0.004 | 331 | 21.2 | L SLF | 0.02 | 784 | 28.8 |
| L SLF | 0.02 | 751 | 27.6 | L SLF | 0.03 | 882 | 32.4 | Fornix | 0.002 | 112 | 20.0 | R ILF | 0.01 | 490 | 26.3 |
| R ATR | 0.004 | 316 | 25.5 | L ATR | 0.003 | 395 | 25.3 | L CB | 0.009 | 141 | 16.6 | L ATR | 0.01 | 306 | 19.6 |
| L ATR | 0.003 | 389 | 24.9 | R ATR | 0.003 | 313 | 25.2 | L SLF | 0.01 | 262 | 9.6 | R ATR | 0.008 | 219 | 17.7 |
| Fornix | 0.001 | 112 | 20.0 | Fornix | 0.001 | 112 | 20.0 | R CST | 0.005 | 316 | 4.7 | R CST | 0.008 | 343 | 5.1 |
| R CST | 0.01 | 213 | 3.2 | R CST | 0.02 | 269 | 4.0 | L CST | 0.006 | 293 | 4.0 | L CST | 0.009 | 171 | 2.3 |
| L CST | 0.006 | 94 | 1.3 | L CST | 0.008 | 197 | 2.7 | L ILF |  |  |  | Fornix |  |  |  |
| **Adjusted tract results** | | | | | | | | | | | | | | | |
| **RD bvFTD>Controls** | | | | **TR bvFTD>Controls** | | | | **AX bvFTD>Controls** | | | | **FA bvFTD<Controls** | | | |
| Tract | p-value | Voxels | % Voxels | Tract | p-value | Voxels | % Voxels | Tract | p-value | Voxels | % Voxels | Tract | p-value | Voxels | % Voxels |
| R UF | 0.03 | 17 | 4.3 | R UF | 0.03 | 9 | 2.3 | L UF | 0.003 | 379 | 58.0 | CC | 0.04 | 875 | 5.9 |
| R ILF | 0.03 | 8 | 0.4 | R ILF | 0.04 | 3 | 0.2 | R UF | 0.008 | 198 | 50.5 | R UF | 0.04 | 17 | 4.3 |
| L UF | 0.05 | 1 | 0.2 | L ILF |  |  |  | CC | 0.01 | 4843 | 32.5 | L UF | 0.04 | 11 | 1.7 |
| L ILF |  |  |  | R SLF |  |  |  | R ILF | 0.02 | 526 | 28.2 | L ATR | 0.05 | 1 | 0.1 |
| R SLF |  |  |  | L SLF |  |  |  | Fornix | 0.005 | 111 | 19.9 | R ILF |  |  |  |
| L SLF |  |  |  | L UF |  |  |  | L ATR | 0.007 | 276 | 17.7 | L ILF |  |  |  |
| R ATR |  |  |  | R ATR |  |  |  | R SLF | 0.01 | 385 | 16.2 | R SLF |  |  |  |
| L ATR |  |  |  | L ATR |  |  |  | R ATR | 0.03 | 138 | 11.1 | L SLF |  |  |  |
| R CB |  |  |  | R CB |  |  |  | L CB | 0.01 | 72 | 8.5 | R ATR |  |  |  |
| L CB |  |  |  | L CB |  |  |  | L SLF | 0.02 | 151 | 5.6 | R CB |  |  |  |
| R CST |  |  |  | R CST |  |  |  | R CST | 0.01 | 156 | 2.3 | L CB |  |  |  |
| L CST |  |  |  | L CST |  |  |  | L CST | 0.01 | 143 | 1.9 | R CST |  |  |  |
| CC |  |  |  | CC |  |  |  | R CB | 0.05 | 1 | 0.5 | L CST |  |  |  |
| Fornix |  |  |  | Fornix |  |  |  | L ILF |  |  |  | Fornix |  |  |  |

**Supplementary Table I.** Summary of diffusivity data by diffusivity metric and region of interest comparing bvFTD with healthy controls. Data displayed are both unadjusted (top) and adjusted for the global mean value for the metric. Blank cells indicate no significant results identified. Key: RD, Radial Diffusivity; TR, Trace Diffusivity; AX, Axial Diffusivity; FA, Fractional Anisotrophy; ATR, Anterior thalamic radiation; CB, cingulum bundle; CC, Corpus Callosum; CST, Corticospinal tract; ILF, Inferior longitudinal fasciculus; SLF, Superior longitudinal fasciculus; UF, Uncinate fasciculus; L, left; R, right.

| **Unadjusted tract results** | | | | | | | | | | | | | | | | | | | |
| --- | --- | --- | --- | --- | --- | --- | --- | --- | --- | --- | --- | --- | --- | --- | --- | --- | --- | --- | --- |
| RD bvFTD>AD | | | | TR bvFTD>AD | | | | FA bvFTD<AD | | | |  |  |  |  |  |  |  |  |
| Tract | p-value | Voxels | % Voxels | Tract | p-value | Voxels | % Voxels | Tract | p-value | Voxels | % Voxels |  |  |  |  |  |  |  |  |
| R UF | 0.002 | 370 | 94.4 | R UF | 0.006 | 338 | 86.2 | R UF | 0.004 | 350 | 89.3 |  |  |  |  |  |  |  |  |
| L UF | 0.003 | 588 | 89.9 | L UF | 0.006 | 550 | 84.1 | L UF | 0.004 | 563 | 86.1 |  |  |  |  |  |  |  |  |
| L CB | 0.003 | 524 | 61.8 | R CB | 0.008 | 417 | 49.2 | R CB | 0.004 | 585 | 69.0 |  |  |  |  |  |  |  |  |
| CC | 0.007 | 7588 | 51.0 | CC | 0.008 | 74 | 40.4 | L CB | 0.006 | 8908 | 59.8 |  |  |  |  |  |  |  |  |
| R CB | 0.003 | 81 | 44.3 | L ATR | 0.02 | 465 | 37.5 | CC | 0.004 | 89 | 48.6 |  |  |  |  |  |  |  |  |
| R ATR | 0.01 | 441 | 35.6 | L CB | 0.01 | 4959 | 33.3 | R ATR | 0.009 | 1054 | 44.3 |  |  |  |  |  |  |  |  |
| R ILF | 0.008 | 615 | 33.0 | R SLF | 0.01 | 593 | 31.8 | R SLF | 0.01 | 777 | 41.7 |  |  |  |  |  |  |  |  |
| R SLF | 0.01 | 647 | 27.2 | R ILF | 0.01 | 454 | 17.2 | L CST | 0.01 | 1023 | 37.6 |  |  |  |  |  |  |  |  |
| Fornix | 0.008 | 124 | 22.2 | R CST | 0.04 | 89 | 15.9 | L ATR | 0.01 | 412 | 33.2 |  |  |  |  |  |  |  |  |
| L ATR | 0.01 | 333 | 21.3 | L SLF | 0.01 | 230 | 14.7 | R ILF | 0.01 | 661 | 25.1 |  |  |  |  |  |  |  |  |
| L ILF | 0.007 | 470 | 17.8 | L ILF | 0.02 | 691 | 10.3 | L SLF | 0.01 | 345 | 22.1 |  |  |  |  |  |  |  |  |
| L SLF | 0.03 | 376 | 13.8 | R ATR | 0.03 | 187 | 7.9 | L ILF | 0.007 | 1070 | 15.9 |  |  |  |  |  |  |  |  |
| R CST | 0.01 | 904 | 13.5 | Fornix | 0.03 | 479 | 6.5 | Fornix | 0.01 | 911 | 12.4 |  |  |  |  |  |  |  |  |
| L CST | 0.01 | 604 | 8.2 | L CST | 0.04 | 98 | 3.6 | R CST |  |  |  |  |  |  |  |  |  |  |  |
| **Adjusted tract results** | | | | | | | | | | | | | | | | | | | |
| **RD bvFTD>AD** | | | | **TR bvFTD>AD** | | | | **AX bvFTD>AD** | | | | **RD AD>bvFTD** | | | | **TR AD>bvFTD** | | | |
| Tract | p-value | Voxels | % Voxels | Tract | p-value | Voxels | % Voxels | Tract | p-value | Voxels | % Voxels | Tract | p-value | Voxels | % Voxels | Tract | p-value | Voxels | % Voxels |
| R UF | 0.02 | 263 | 67.1 | R UF | 0.02 | 215 | 54.8 | L UF | 0.04 | 107 | 16.4 | L ILF | 0.03 | 289 | 11.0 | L ILF | 0.03 | 736 | 27.9 |
| L UF | 0.01 | 390 | 59.6 | L UF | 0.03 | 237 | 36.2 | R UF | 0.03 | 13 | 3.3 | L SLF | 0.04 | 87 | 3.2 | L SLF | 0.02 | 524 | 19.3 |
| R CB | 0.04 | 52 | 28.4 | R ILF | 0.02 | 306 | 16.4 | R ILF | 0.02 | 3 | 0.2 | CC | 0.03 | 162 | 1.1 | CC | 0.02 | 303 | 2.0 |
| CC | 0.03 | 1919 | 12.9 | L ILF | 0.03 | 123 | 4.7 | CC | 0.05 | 2 | 0.01 | R ILF |  |  |  | L CB | 0.02 | 10 | 1.2 |
| R ATR | 0.03 | 146 | 11.8 | CC | 0.03 | 246 | 1.7 | L ILF |  |  |  | R SLF |  |  |  | R ILF |  |  |  |
| R ILF | 0.01 | 181 | 9.7 | R CST | 0.04 | 88 | 1.3 | R SLF |  |  |  | R UF |  |  |  | R SLF |  |  |  |
| L ILF | 0.02 | 210 | 8.0 | R SLF |  |  |  | L SLF |  |  |  | L UF |  |  |  | R UF |  |  |  |
| R CST | 0.02 | 378 | 5.6 | L SLF |  |  |  | R ATR |  |  |  | R ATR |  |  |  | L UF |  |  |  |
| R SLF | 0.03 | 124 | 5.2 | R ATR |  |  |  | L ATR |  |  |  | L ATR |  |  |  | R ATR |  |  |  |
| L ATR | 0.02 | 80 | 5.1 | L ATR |  |  |  | R CB |  |  |  | R CB |  |  |  | L ATR |  |  |  |
| L CST | 0.02 | 232 | 3.2 | R CB |  |  |  | L CB |  |  |  | L CB |  |  |  | R CB |  |  |  |
| L CB | 0.02 | 10 | 1.2 | L CB |  |  |  | R CST |  |  |  | R CST |  |  |  | R CST |  |  |  |
| Fornix |  |  |  | Fornix |  |  |  | Fornix |  |  |  | L CST |  |  |  | L CST |  |  |  |
| L SLF |  |  |  | L CST |  |  |  | L CST |  |  |  | Fornix |  |  |  | Fornix |  |  |  |

**Supplementary Table II.** Summary of diffusivity data by diffusivity metric and region of interest comparing bvFTD with Alzheimer’s disease. Data displayed are unadjusted (top) and adjusted for the global mean value for the metric. Blank cells indicate no significant results identified. Each DTI metric was contrasted in both directions only results surviving FWE correction p<0.05 are displayed above. . Key: RD, Radial Diffusivity; TR, Trace Diffusivity; AX, Axial Diffusivity; FA, Fractional Anisotrophy; ATR, Anterior thalamic radiation; CB, cingulum bundle; CC, Corpus Callosum; CST, Corticospinal tract; ILF, Inferior longitudinal fasciculus; SLF, Superior longitudinal fasciculus; UF, Uncinate fasciculus; L, left; R, right.

|  |  |  |  |  |  |  |  |  |  |  |  |  |  |  |  |  |  |  |  |
| --- | --- | --- | --- | --- | --- | --- | --- | --- | --- | --- | --- | --- | --- | --- | --- | --- | --- | --- | --- |
| **MAPT>CONT RD** | | | | **MAPT>CONT TR** | | | | **MAPT>CONT AX** | | | | **MAPT<CONT FA** | | | | **C9>CONT AX** | | | |
| Tract | p-value | Voxels | %Voxels | Tract | p-value | Voxels | %Voxels | Tract | p-value | Voxels | %Voxels | Tract | p-value | Voxels | %Voxels | Tract | p-value | Voxels | %Voxels |
| L UF | 0.008 | 53 | 8.1 | L UF | 0.009 | 37 | 5.7 | Fornix | 0.004 | 123 | 22.0 | L UF | 0.01 | 62 | 9.5 | CC | 0.04 | 278 | 1.9 |
| L ILF | 0.01 | 82 | 1.6 | L ILF | 0.01 | 59 | 2.2 | L UF | 0.006 | 275 | 42.0 | Fornix | 0.02 | 169 | 30.2 | L CB | 0.03 | 32 | 3.8 |
| Fornix | 0.05 | 9 | 1.6 | Fornix | 0.04 | 49 | 8.8 | R UF | 0.008 | 180 | 45.9 | L ILF | 0.02 | 130 | 4.9 |  |  |  |  |
|  |  |  |  | CC | 0.04 | 21 | 0.1 | L ATR | 0.009 | 140 | 9.0 | R ATR | 0.02 | 21 | 1.7 |  |  |  |  |
|  |  |  |  |  |  |  |  | R ATR | 0.02 | 13 | 1.0 | L ATR | 0.02 | 12 | 0.8 |  |  |  |  |
|  |  |  |  |  |  |  |  | L CB | 0.02 | 90 | 10.6 |  |  |  |  |  |  |  |  |
|  |  |  |  |  |  |  |  | CC | 0.03 | 2477 | 16.6 |  |  |  |  |  |  |  |  |
|  |  |  |  |  |  |  |  | L CST | 0.03 | 106 | 1.4 |  |  |  |  |  |  |  |  |
|  |  |  |  |  |  |  |  | L SLF | 0.03 | 52 | 1.9 |  |  |  |  |  |  |  |  |
|  |  |  |  |  |  |  |  | L ILF | 0.03 | 13 | 0.5 |  |  |  |  |  |  |  |  |
|  |  |  |  |  |  |  |  | R ILF | 0.03 | 184 | 9.9 |  |  |  |  |  |  |  |  |
|  |  |  |  |  |  |  |  | R CB | 0.05 | 7 | 3.8 |  |  |  |  |  |  |  |  |
| **MAPT>AD RD** | | | | **MAPT>AD TR** | | | | **MAPT>AD AX** | | | |  |  |  |  |  |  |  |  |
| Tract | p-value | Voxels | %Voxels | Tract | p-value | Voxels | %Voxels | Tract | p-value | Voxels | %Voxels |  |  |  |  |  |  |  |  |
| L ILF | 0.02 | 209 | 7.9 | L ILF | 0.02 | 188 | 7.1 | L ILF | 0.04 | 24 | 0.9 |  |  |  |  |  |  |  |  |
| L UF | 0.02 | 136 | 20.8 | R UF | 0.03 | 105 | 26.8 |  |  |  |  |  |  |  |  |  |  |  |  |
| R UF | 0.03 | 44 | 11.2 | L UF | 0.03 | 277 | 42.4 |  |  |  |  |  |  |  |  |  |  |  |  |
| R ILF | 0.03 | 84 | 4.5 | R ILF | 0.03 | 200 | 10.7 |  |  |  |  |  |  |  |  |  |  |  |  |
| Fornix | 0.04 | 145 | 25.9 | CC | 0.04 | 172 | 1.2 |  |  |  |  |  |  |  |  |  |  |  |  |
| R ATR | 0.04 | 35 | 2.8 | L ATR | 0.05 | 25 | 1.6 |  |  |  |  |  |  |  |  |  |  |  |  |
| CC | 0.05 | 92 | 0.6 |  |  |  |  |  |  |  |  |  |  |  |  |  |  |  |  |
| R CST | 0.05 | 11 | 0.2 |  |  |  |  |  |  |  |  |  |  |  |  |  |  |  |  |

**Supplementary Table III.** Summary of diffusivity metrics comparing genetic subgroups with healthy controls and Alzheimer’s disease.

Data displayed are adjusted for the global mean value of the metric. DTI metric was contrasted in both directions only results

surviving FWE correction p<0.05 are displayed above.
